# The spiritual core of the hard problem: consciousness as foundational, not emergent

**DOI:** 10.3389/fpsyg.2025.1659944

**Published:** 2025-09-04

**Authors:** Amira Arora

**Affiliations:** Independent Researcher, Trenton, NJ, United States

**Keywords:** consciousness is primary, transpersonal psychology, non-dual traditions, Advaita Vedanta, contemplative science, participatory knowing, spiritual phenomenology, cosmology and consciousness

## Abstract

This paper proposes a transpersonal reframing of the Hard Problem of Consciousness by positing that consciousness is ontologically primary—not an emergent property of neural processes, but the foundational reality from which mind and matter arise. Integrating insights from non-dual spiritual traditions such as Advaita Vedanta and Tibetan Buddhism, contemplative science, and the work of transpersonal theorists including Jorge Ferrer, Ken Wilber, and Stanislav Grof, the study argues that a consciousness-centered metaphysics offers a more coherent model for explaining subjectivity, intentionality, and qualia. In critiquing materialist reductionism, it highlights the limitations of third-person methodologies and emphasizes the legitimacy of first-person and participatory ways of knowing. The paper also explores the broader epistemological, ethical, cultural, and ecological implications of adopting a transpersonal cosmology—one that bridges science and spirituality without collapsing their distinctions. By shifting the ontological center from matter to consciousness, this framework invites a pluralistic, integrative paradigm for understanding reality, advancing both human flourishing and scientific inquiry.

## Introduction

The so-called “hard problem of consciousness,” famously articulated by philosopher David Chalmers (1995), asks why and how physical processes in the brain give rise to subjective experience. Why does neural activity in the visual cortex generate the qualia of red, or neurochemical shifts involving oxytocin result in the felt sense of love? Despite significant advances in cognitive neuroscience, this explanatory gap remains unresolved (Koch et al., 2016). Consciousness, the very medium through which experience arises, continues to resist capture within strictly reductive or materialist paradigms (Kelly et al., 2015). Transpersonal psychology, rooted in an expanded ontology that embraces the spiritual dimension of human life, offers a compelling framework for reimagining consciousness beyond mechanistic assumptions. This paper proposes that consciousness is not a byproduct of brain activity but rather ontologically fundamental. In this view, the brain acts as a filter or interface for a more pervasive, nonlocal consciousness: a model echoed in contemplative traditions, spiritual phenomenology, Indigenous epistemologies, and postmaterialist research (Tart, 2009; Greyson, 1983; Beauregard, 2014). Far from dismissing scientific inquiry, this perspective seeks to enrich it through integration with millennia of contemplative practice, cross-cultural wisdom, and first-person methodologies (Vieten et al., 2013).

## The materialist paradigm and its limits

The dominant scientific framework for understanding consciousness remains grounded in materialism, or physicalism, which holds that consciousness emerges from complex neurobiological interactions. According to this paradigm, the brain is the sole generator of subjective experience. However, despite decades of research in cognitive neuroscience, no empirical mechanism has been identified that explains how neuronal processes give rise to *qualia*—the ineffable textures of experience such as the redness of red or the warmth of compassion (Chalmers, 2020). While neuroimaging studies have established correlations between brain states and subjective reports, correlation is not causation. As many philosophers of mind have argued, these correlations fail to bridge the explanatory gap: one cannot deduce the qualitative content of experience from neural data alone (Nagel, 1974; Levine, 1983).

Materialist accounts also struggle to assimilate insights from physics, particularly quantum mechanics and systems theory, which increasingly suggest a participatory universe. The observer effect in quantum experiments, whereby the outcome appears dependent on the act of measurement, raises profound questions about the role of consciousness in shaping reality (Rosenblum and Kuttner, 2011; Stapp, 2017). While such claims are often overstated in popular science, careful interpretations indicate that consciousness may not be an epiphenomenon of matter, but rather a fundamental component of it: an idea congruent with panpsychism, dual-aspect monism, and idealism (Goff, 2019; Kastrup, 2020). Systems theory and non-linear dynamics also point to emergent phenomena and self-organizing systems that challenge reductionist assumptions, opening space for more integrative models of mind and matter (Capra and Luisi, 2014).

In addition, materialist models struggle to accommodate empirical reports of transpersonal experiences—such as near-death experiences (NDEs), shared death experiences, veridical out-of-body experiences, and spontaneous mystical states—which often occur under conditions of significantly diminished or absent brain function (Greyson, 1983; Lommel, 2010). Neuroimaging studies of psychedelics have also shown that reduced activity in certain brain regions (especially the default mode network) corresponds to heightened subjective richness and spiritual unity, contrary to expectations of materialist predictions (Carhart-Harris et al., 2014). These findings suggest that the brain may act more as a filter or receiver of consciousness, rather than its producer, reviving older models like the transmission or filter theory proposed by thinkers such as William James and Henri Bergson (Kelly et al., 2015).

Additionally, materialism provides little explanatory power for why certain ordinary or spontaneous states, such as awe in nature, meditative absorption, or sudden feelings of bliss and sacredness, carry an ineffable, often transformative quality. These experiences are increasingly recognized not as fringe anomalies, but as integral to psychological resilience and human flourishing (Garcia-Romeu et al., 2015). William James (1902/2002), in his seminal work *The* Var*ieties of Religious Experience*, argued that mystical and spiritual experiences reveal a more expansive layer of consciousness that is foundational to human identity and meaning-making. This dimension, long neglected by materialist models, is being reintroduced into mainstream psychology through the growing fields of transpersonal and contemplative science (Vieten et al., 2013).

## The transpersonal reframing: consciousness as primary

Transpersonal psychology, emerging from the work of Abraham Maslow, Stanislav Grof, and later theorists such as Ken Wilber, posits that human consciousness possesses dimensions that transcend the individual ego and material body. Rather than being confined to neuronal processes, consciousness is seen as ontologically fundamental—a dimension of reality itself. Jorge Ferrer (2002) critiques the individualist and representational bias in classical models and introduces a participatory paradigm, wherein transpersonal knowing arises through embodied and relational engagement with a multidimensional reality. In this reframing, consciousness is not merely subjective experience produced by the brain, but a participatory field in which minds, bodies, and the world interpenetrate and co-emerge (Ferrer and Sherman, 2008).

This view resonates with non-Western philosophical traditions, particularly Advaita Vedanta, which asserts that consciousness (Brahman) is the sole reality and the empirical world is Maya—an illusory appearance (Shankara, n.d.). The *Mandukya Upanishad* proclaims, “All this is indeed Brahman. This Self is Brahman.” In this model, the individual self (Atman) is not distinct from the universal consciousness, it is a localized, conditioned expression of it. The brain and body, therefore, do not produce consciousness but serve as filters, modulators, or receivers of it—a notion increasingly supported by contemporary neuroscience of psychedelics and NDEs (Carhart-Harris et al., 2014; Martial et al., 2020; Greyson, 2021).

Parallel views can be found in Tibetan Buddhist Dzogchen and Mahamudra teachings, which describe the mind’s essence as *rigpa*—a self-knowing, luminous awareness that is empty of inherent form yet inherently awake. These traditions emphasize *direct realization* through meditation rather than philosophical speculation, pointing to consciousness not as a metaphysical abstraction but as an immediate, experiential ground of being. The phenomenological consonance between these teachings and non-dual awareness reported in Western contemplative and psychedelic research (Millière et al., 2018; Letheby, 2021) underscores the cross-cultural consistency of consciousness as foundational.

Likewise, mystics across traditions—from Meister Eckhart and Teresa of Avila to Ramana Maharshi and Sri Aurobindo—have described states of awareness in which the usual subject-object duality collapses. These unique experiences reveal a non-dual ground of being that is both ineffable and intrinsically meaningful. Rather than dismissing such experiences as pathological or anomalous, transpersonal psychology regards them as windows into the nature of reality itself. Empirical studies on non-dual awareness and mystical states increasingly support this view (Garcia-Romeu et al., 2015), especially in clinical contexts where these experiences catalyze post-traumatic growth and existential transformation.

Ken Wilber’s Integral Theory expands this framework by portraying consciousness as the apex of an evolutionary spectrum moving from matter to body to mind to soul to spirit. His AQAL (All Quadrants, All Levels) model synthesizes developmental psychology, spirituality, and systems theory into a comprehensive paradigm, suggesting that the cosmos is not only conscious but evolving toward ever-greater integration and awareness (Wilber, 2017). This integrative vision is echoed by contemporary thinkers like Thomas Metzinger (2020) and Thompson (2021), who, despite differing ontologies, explore consciousness in a relational and dynamic light. Together, these approaches reinforce the view that consciousness is not a byproduct of the brain but a fundamental feature of existence—one that can no longer be ignored in psychological science.

## The filter theory and transpersonal consciousness

One influential model that integrates transpersonal insights with contemporary scientific perspectives is the **filter theory** of consciousness. Originally suggested by thinkers such as James (1898/2002), Huxley (1954a), and Huxley (1954b), this theory posits that the brain does not generate consciousness but functions as a filter, receiver, or reducing valve. In this analogy, just as a radio tunes into and selects specific frequencies from the broad spectrum of electromagnetic waves, the brain filters and constrains a vast, undifferentiated field of consciousness into a form adapted for practical survival and everyday functioning within the material world (James, 1898/2002; Huxley, 1954a; Huxley, 1954b).

More recently, this idea has been revitalized and elaborated upon by philosophers and transpersonal psychologists. Kastrup (2020), in his analytic idealism framework, argues that mind, rather than matter, constitutes the fundamental ontological category, with the brain acting as a complex filter that shapes conscious experience. Similarly, Stanislav Grof’s extensive research on holotropic states highlights how consciousness transcends the personal ego and physical body, extending into archetypal, ancestral, and even cosmological dimensions of awareness (Grof, 1985; Grof and Grof, 2017). Grof’s model situates the brain as an interface that modulates access to these transpersonal domains based on physiological and psychological states.

Empirical research supports this filter model in several ways. Neuroimaging studies of psychedelic states reveal decreased activity in the brain’s default mode network, correlated with a loosening of ego boundaries and expansive conscious experience (Carhart-Harris et al., 2018). Near-death experience (NDE) research similarly suggests that consciousness can persist or even intensify despite significant reductions or alterations in brain activity (Timmermann et al., 2021; Greyson, 2021). These findings challenge the classical materialist assumption that consciousness is strictly contingent on normal brain functioning and instead align with the filter hypothesis.

The filter theory also gains tentative support from psi research investigating phenomena such as precognition, telepathy, and remote viewing (Radin, 2006). While controversial, such findings, when carefully controlled, suggest that consciousness may not be entirely confined by classical space–time limitations, further destabilizing reductionist assumptions. If consciousness indeed extends beyond the brain and body, then understanding its nature requires integrating these anomalous phenomena rather than dismissing them outright.

Importantly, this model explains why non-ordinary states of consciousness, whether induced by trauma, meditation, psychedelics, or mystical absorption, often feel subjectively “more real” or authentic than ordinary waking consciousness (Greyson, 1983; Vago and Zeidan, 2016). The filter, when relaxed, allows access to a broader spectrum of awareness that is normally constrained by neurobiological and cognitive limits. Contrary to the assumption that reduced neural activity corresponds to diminished consciousness, many such states demonstrate a paradoxical intensification of conscious experience.

Cross-cultural spiritual traditions corroborate this perspective. Indigenous epistemologies frequently describe consciousness as an expansive field accessible through altered states, with the physical body serving as a limited conduit (Cajete, 2000; Kimmerer, 2013). Similarly, Eastern contemplative traditions emphasize the modulation rather than the creation of awareness by the body–mind system (Wallace, 2007; Lutz et al., 2019). This convergence of modern science and ancient wisdom lends credence to the filter model as a robust framework for reconceptualizing consciousness beyond the materialist paradigm.

## Phenomenology and the intimacy of experience

Transpersonal psychology places significant emphasis on **first-person phenomenology**—the rigorous, introspective study of subjective experience—as an essential approach to understanding consciousness. The deeply subjective nature of qualia, or what-it-is-like aspects of experience, cannot be fully captured through third-person scientific observation alone (Chalmers, 1995; Varela, 1996). This epistemological insight underscores why spiritual practices such as meditation, prayer, and mindfulness are not merely therapeutic interventions but systematic methods for investigating the fundamental nature of mind and reality.

Contemplative traditions across cultures provide detailed phenomenological maps that describe progressive transformations in identity, perception, and intentionality. In Tibetan Buddhism, for example, the sequential stages of *shamatha* (calm abiding) and *vipassana* (insight) meditation lead practitioners to the direct experiential realization of the “nature of mind,” which is often described as luminous, empty, and self-knowing (Wallace, 2007; Lutz et al., 2019). These states transcend conceptual thought and evoke a mode of awareness that is both non-dual and primordial. Similarly, advanced practitioners of Transcendental Meditation (TM) report encounters with **pure consciousness**—a contentless, yet fully present awareness—that suggests an ontologically basic level of mind distinct from ordinary waking cognition (Travis and Shear, 2010; Metzinger, 2020).

Crucially, such non-ordinary experiences appear consistently across diverse cultures and historical periods, reflecting a potentially universal human capacity to access consciousness beyond the confines of egoic selfhood and neural activity (Ferrer, 2017; Hartelius and Ferrer, 2020). These states are not pathological or hallucinatory distortions but are often correlated with enduring psychological benefits, including enhanced clarity, compassion, emotional integration, and resilience. Such qualities align with core markers of psychological health within the transpersonal framework (Walsh and Vaughan, 1993; Baer, 2003).

Recent advances in contemplative neuroscience provide empirical support for these phenomenological insights. Long-term meditation practitioners exhibit increased high-amplitude gamma oscillations, particularly in prefrontal cortical regions associated with attention and emotional regulation (Lutz et al., 2004). Structural neuroimaging studies reveal cortical thickening and enhanced connectivity in areas implicated in meta-awareness and self-regulation (Fox et al., 2014). However, these neural correlates, while informative, do not exhaustively explain consciousness itself; rather, they illustrate how disciplined contemplative practice can transform the brain’s function and structure while affirming the primacy of subjective experience as the irreducible core of consciousness (Vago and Zeidan, 2016).

Moreover, phenomenological methodologies, such as micro-phenomenology and neurophenomenology, are bridging first-person accounts with third-person scientific data, enriching our understanding of consciousness as a dynamic, embodied process (Petitmengin, 2006; Varela et al., 2016). This integrative approach challenges reductive materialism and supports a more holistic science that honors the **intimacy of experience** without losing rigor.

## Addressing objections and the need for epistemic humility

Critics of the non-materialist view often argue that without direct empirical evidence, proposing consciousness as fundamental amounts to metaphysical speculation. However, this criticism assumes that metaphysical naturalism is the only valid philosophical position. As Ferrer (2002) and others have pointed out, the dominance of scientific materialism itself rests on metaphysical assumptions rather than proven facts. Every worldview inevitably makes basic foundational commitments that cannot be empirically verified.

If we take subjective experience seriously, as something real rather than an illusion or mere byproduct, a consciousness-centered model becomes more straightforward. It does not have to explain how non-conscious matter generates awareness; instead, it views matter as a manifestation or modulation of mind. Although this shift may seem radical, it is not opposed to science. Rather, it calls for a broader approach to knowledge, one that values both first-person insight and third-person observation. This perspective demands epistemic humility. The nature of consciousness might never be fully explained by objective methods alone. As transpersonal psychologist Washburn (1995) observes, the psyche contains depths beyond the reach of egoic awareness. Spiritual traditions have long recognized this, emphasizing contemplative practice not only for healing but for deep ontological understanding.

Moreover, it is important to acknowledge the complexity and diversity within foundational models of consciousness. Not every mystical or transpersonal experience leads to the same metaphysical conclusion. Interpretations differ widely depending on cultural, linguistic, and doctrinal backgrounds. Rather than a weakness, this diversity reflects the richness of human engagement with consciousness.

## Toward a transpersonal epistemology and future directions

Reframing consciousness as ontologically foundational opens not only new theoretical vistas but also profound epistemological and practical implications. If consciousness is primary, then the methodologies we use to investigate reality must expand beyond conventional third-person empirical inquiry to include first-person experiential methods, introspective phenomenology, and contemplative wisdom. Ferrer’s (2002, 2017) participatory epistemology provides a vital framework here, emphasizing that knowing arises through embodied, relational engagement with a multidimensional and co-creative cosmos. This approach invites the cultivation of inner capacities such as mindfulness, intuition, and contemplative awareness—not solely for accessing transient altered states but for stabilizing enduring insights that illuminate the deeper nature of being and reality.

Such a transpersonal epistemology challenges the rigid dichotomy between subject and object, science and spirituality, and opens avenues for integrative approaches across education, psychotherapy, and healthcare. Contemplative pedagogy, for instance, nurtures whole-person learning by incorporating mindfulness and introspective practices, fostering not only cognitive understanding but transformative wisdom (Hartelius and Ferrer, 2020; Lutz et al., 2019). In psychotherapy, integrating transpersonal perspectives can deepen healing by recognizing expanded states of consciousness as authentic dimensions of human experience rather than symptoms to suppress (Grof and Grof, 2017; Lukoff et al., 1998). Integrative medicine similarly benefits by acknowledging subtle energetic and spiritual aspects of health, reflecting an embodied and participatory worldview (Kelly et al., 2015).

Importantly, this expanded epistemology embraces mystery and the sacred as essential to scientific inquiry rather than obstacles to be eliminated (Ferrer, 2017). It opens space for a multidimensional science that respects the lived, subjective reality of consciousness while maintaining rigorous inquiry through interdisciplinary dialogue (Varela et al., 2016). By valuing spiritual insight alongside empirical data, this approach fosters a more holistic and inclusive understanding of mind, body, and world.

## Cultural implications and the need for a new metaphysical narrative

The implications of adopting a consciousness-centered ontology extend far beyond academic theory, reaching deeply into cultural and civilizational domains. Our prevailing metaphysical narrative, anchored in scientific materialism and mechanistic paradigms, has contributed to widespread alienation, ecological destruction, and a profound crisis of meaning and belonging (Berry, 1988; Macy, 2013). This story reduces the Earth and other beings to inert resources, thereby fueling exploitation and disconnection. Reframing consciousness as the foundational ground enables a radical renewal of values centered on sacredness, interconnectedness, and reverence for life itself. Indigenous epistemologies provide powerful examples here, emphasizing relationality and the Earth as a living, sentient subject imbued with spirit (Cajete, 2000; Kimmerer, 2013; Santos-Granero, 2018). These perspectives resist the Cartesian split between human and nature, underscoring that consciousness and life are co-creative and interdependent processes.

Thinkers like Thomas Berry (1988) and Macy (2013) describe this needed cultural transformation as the “Great Turning”: a shift from an industrial growth-oriented society to an ecological civilization grounded in respect, reciprocity, and ecological wisdom. In this emerging worldview, consciousness is not private or isolated but fundamentally relational and ecological, embedded within webs of life that include humans, nonhuman beings, and the Earth itself. Ethics expand accordingly, moving from control and domination toward communion, care, and stewardship (Kimmerer, 2013; Ferrer, 2017).

Thus, embracing consciousness as foundational is not merely an intellectual exercise but a call to reimagine our place in the cosmos and our responsibilities to one another and the planet. It invites a new metaphysical narrative that can inspire sustainable living, social justice, and spiritual flourishing in the Anthropocene and beyond.

## Cross-cultural perspectives on primordial consciousness

Across a wide array of spiritual and cultural traditions, the experience of expanded, unitary, or non-dual consciousness is a recurring and striking motif. In the Upanishads, **ātman** is equated with **Brahman**, revealing an identity between individual consciousness and the ultimate reality. Sufi mystics like Rumi speak of annihilation of the self (*fanā’*) into divine love, mirroring Buddhist notions of egoless awareness (*anattā*) and Christian mystics’ descriptions of union with God (Underhill, 2002). Indigenous shamanic cosmologies, too, often center on the permeability between spirit, self, and nature, underscoring a non-dualistic cosmology in which consciousness is not isolated to the human but distributed across the living world (Eliade, 2004; Harvey, 2005; Kopenawa and Albert, 2013).

Despite doctrinal and cultural differences, these diverse traditions share a phenomenological pattern: encounters with a deeper field of being, often described as luminous, infinite, and ineffable. Transpersonal scholars have termed this shared motif a *perennial thread*, not in the sense of a universal dogma, but as a convergence of direct experiential insight into the nature of consciousness and reality (Huxley, 1945; Wilber, 2000; Ferrer, 2002). These cross-cultural resonances lend credence to the hypothesis that consciousness is not merely a culturally constructed or neurochemically generated epiphenomenon, but a fundamental and perhaps ontologically real dimension of existence.

This coherence across traditions challenges both pathological and relativistic interpretations of spiritual experiences common in reductionist frameworks. Rather than viewing mystical states as cognitive distortions or culturally specific illusions, these experiences may reflect authentic insights into a participatory cosmos accessible through diverse epistemic lineages (Ferrer, 2017; Hartelius et al., 2013). Ferrer’s notion of *epistemological pluralism* becomes essential here: it posits that different cultures and spiritual traditions offer unique but complementary access points to a multidimensional reality. This pluralism resists both colonial universalism and radical relativism by emphasizing co-validity rather than hierarchy or equivalence among traditions.

Additionally, modern contemplative neuroscience is beginning to map neural correlates of these expanded states, such as decreased default mode network activity and increased gamma coherence, suggesting that spiritual practices are not anomalous but measurable and trainable capacities of consciousness (Lutz et al., 2004; Josipovic, 2014). These findings offer empirical bridges between ancient wisdom and contemporary science, inviting integrative frameworks that honor both subjective depth and objective rigor.

## A challenge to scientific orthodoxy

Reclaiming consciousness as ontologically primary directly confronts the metaphysical underpinnings of modern science. While science remains an indispensable and powerful epistemic method, it has long been tethered to the assumptions of physicalism and reductionism, which define consciousness as an emergent byproduct of complex neural computation. This stance, however, remains deeply contested and arguably incomplete, especially in light of the enduring “hard problem” of consciousness—namely, the challenge of explaining how subjective experience arises from objective brain processes (Chalmers, 1995).

The integration of contemplative and transpersonal approaches expands the scientific project into a broader, more inclusive epistemology, one capable of investigating the full spectrum of consciousness, including its non-ordinary and transformative dimensions. This shift is increasingly being championed by philosophers and scientists who are revisiting ancient ideas in light of contemporary evidence. Thinkers such as Strawson (2006) and Goff (2019), have revitalized interest in **panpsychism** and **cosmopsychism**, positions that view consciousness as a fundamental, ubiquitous aspect of the universe, rather than a localized anomaly of human brains.

Similarly, idealist and non-dualist metaphysical models, long marginalized within academic discourse, are gaining renewed attention for their explanatory power. Bernardo Kastrup (2020), for example, proposes analytic idealism, suggesting that all physical reality arises from patterns of consciousness, a view that converges with classical Vedanta and aspects of Mahayana Buddhism. These views challenge the epistemic hegemony of materialism and open the door to interdisciplinary dialogue across philosophy, physics, neuroscience, and contemplative traditions (Varela et al., 2016; Jonas, 2016).

By moving beyond the inherited metaphysics of control and objectivity, this emerging paradigm makes room for a science that is participatory, reflexive, and open to the sacred. It urges us to reexamine not just what we know, but *how* we come to know—and who we become in the process. As such, a transpersonal vision of science offers not only epistemological expansion but ethical and existential depth, suggesting that the inquiry into consciousness may ultimately be inseparable from the transformation of the knower.

## Existential and ethical reflections

If consciousness is foundational, then the moral fabric of reality is transformed. Every being, and potentially every phenomenon in the cosmos, merits intrinsic reverence. Ethics, from this standpoint, is not merely the product of social contract theory or utilitarian reasoning, but an outgrowth of ontological intimacy: the realization that all entities arise within and as expressions of a unified conscious field. This resonates with the ethical implications found in non-dual traditions, where the perception of separateness dissolves and compassion becomes spontaneous (Wilber, 2000; Josipovic, 2014).

The spiritual realization of non-duality does not encourage withdrawal from the world but grounds engagement in humility, care, and service. As contemplative traditions across cultures affirm, awakening to a deeper stratum of being tends to foster an ethos of interconnection and moral responsibility (Tolle, 2005; Kabat-Zinn, 2003). In this sense, spiritual realization becomes an ethical imperative, particularly in the context of ecological degradation, systemic injustice, and cultural fragmentation.

The transpersonal perspective offers not merely psychological relief but existential orientation. In an age marked by anxiety, isolation, and meaninglessness, reframing consciousness as primary provides a pathway toward wholeness—a remembrance of embeddedness within a sacred, living cosmos. Meaning is no longer externally imposed or constructed in the aftermath of nihilism; it is discovered in the depths of conscious presence itself. This reframing has the potential to recalibrate not only individual lives but collective values, inviting a cultural ethos grounded in reverence, presence, and co-flourishing.

## Ontological reversals and participatory realism

Recent developments in philosophy of mind and consciousness studies suggest a growing openness to models that invert the traditional ontological order. Building on phenomenology and quantum theory, argues that consciousness cannot be derived from material conditions because it is the precondition of all knowing. In his view, consciousness is not located within space and time but is the *a priori* condition for space–time’s very appearance. Thompson (2015), informed by Buddhist philosophy and cognitive science, articulates an “enactive” framework in which mind, body, and world co-arise in a process of dynamic interdependence. This intersubjective and embodied model challenges Cartesian dualism and supports a more fluid, emergent understanding of consciousness.

These insights find resonance in Ferrer’s (2002) participatory realism, which maintains that spiritual realities are not fixed metaphysical entities but are enacted through participatory engagement. According to this view, mystical and transpersonal experiences are not reducible to subjective illusions, nor are they revelations of pre-existing absolutes. Instead, they are co-arisen events, emergent phenomena that result from the intersection of personal intention, cultural-symbolic systems, and deeper ontological potentials.

This participatory framework avoids the extremes of metaphysical relativism and naïve realism. It allows us to take spiritual and transpersonal experiences seriously without claiming final or absolute authority for any one interpretation. The cosmos, from this perspective, is not a closed system but a co-evolving field of becoming—responsive to intention, capable of surprise, and suffused with sacred potential (Ferrer, 2017; Kelly et al., 2015). Rather than viewing mystical insight as hallucinatory or fantastical, participatory realism treats it as a kind of ontological dialogue, wherein human consciousness and the greater field of being interpenetrate to generate meaning and manifestation.

Such a vision has profound implications. It suggests that the structure of reality is neither inert nor indifferent but is permeable to consciousness itself. Spiritual realization is no longer a retreat from the world, but an act of world-making, a co-creative engagement with the deeper currents of existence. This model reorients metaphysics around communion rather than control and points toward an integrative future in which science, spirituality, and culture may converge in a renewed exploration of consciousness and cosmos.

## Toward a transpersonal cosmology

If we take the foundational nature of consciousness seriously, then our cosmology (the story we tell about the universe) must be radically reimagined. In a transpersonal framework, the cosmos is not a cold, mechanistic expanse but a living, evolving field of intelligence. This reframing aligns with traditions such as process theology, articulated by Alfred North Whitehead (1929/1978), which views reality as dynamic and relational, with God as a participatory process unfolding through time. Contemporary theologians such as Keller (2008) have expanded on this vision, emphasizing relationality, creativity, and the sacredness of becoming.

Such a cosmology mirrors the insights of transpersonal psychology: the universe is not inert but awakening. Human beings are not isolated accidents of evolutionary chance but nodal points in an intelligent web of consciousness. This view resonates with Indigenous cosmologies, such as those of the Ainu, Navajo, or Aboriginal Australians, where the cosmos is understood as alive, sentient, and sacred (Cajete, 2000; Atleo, 2011). These traditions have long held that consciousness is not limited to human minds but pervades all aspects of nature.

A transpersonal cosmology also supports a form of deep ecological ethics. If consciousness is ontologically primary, then nature is not a passive resource but a participant in a sacred unfolding. Such a framework challenges anthropocentrism and invites reverence for all life forms. It bridges science and spirituality not by collapsing one into the other, but by recognizing that multiple epistemologies—empirical, contemplative, participatory—can illuminate different dimensions of reality (Ferrer, 2002). This cosmology affirms that meaning, value, and intelligence are embedded within the very fabric of existence.

## Discussion

The arguments presented in this paper call for a fundamental shift in how consciousness is approached within psychology, neuroscience, and the philosophy of mind. Recognizing consciousness as ontologically primary disrupts the materialist orthodoxy that dominates contemporary discourse. Rather than treating subjective experience as a byproduct of neuronal activity, a consciousness-centered ontology places awareness itself at the foundation of reality. This perspective aligns with a growing number of interdisciplinary thinkers who challenge reductive paradigms. Scholars such as Thompson (2015), and Thomas Metzinger (2009) have argued for approaches that integrate phenomenology, enactivism, and contemplative science. Their work underscores that consciousness cannot be adequately explained from a third-person perspective alone. Instead, the first-person dimension—subjective, immediate, and irreducible—must be given equal epistemological weight.

The transpersonal perspective validates the insights of contemplative traditions that have long emphasized inner knowing, spiritual transformation, and non-dual awareness. Far from being idiosyncratic or culturally bounded, the recurring phenomenological structures of mystical experience across traditions suggest a deeper commonality (Hood, 2001). The widespread reports of unity, timelessness, ego dissolution, and sacred presence indicate that such experiences may reveal fundamental aspects of consciousness, not mere neurobiological anomalies.

One of the most important implications of this view is the need for epistemological pluralism. As Ferrer (2002) has argued, knowledge about consciousness should not be limited to detached, third-person observation. Participatory knowing, rooted in transformation, relationship, and experiential depth, is a legitimate and necessary complement. Such pluralism does not undermine scientific integrity but enriches it. Integrative methodologies, such as neurophenomenology (Varela, 1996), first-person science (Depraz et al., 2003), and contemplative inquiry, can broaden psychology’s scope and offer a more complete picture of consciousness.

This framework also invites interdisciplinary collaboration. Philosophers, neuroscientists, anthropologists, theologians, and contemplative practitioners each contribute unique insights into the nature of mind and reality. Future research might include longitudinal studies of spiritual development, neurophenomenological studies of altered states, or comparative analyses of mystical experiences across traditions. The emerging field of contemplative science (Davidson and Goleman, 1977; Dahl et al., 2015) offers promising directions for empirically studying these experiences while honoring their inner depth.

Finally, this consciousness-centered paradigm invites not just intellectual reconsideration but cultural and ethical transformation. A worldview in which consciousness is sacred, participatory, and relational reframes our responsibilities to each other, to the planet, and to the unfolding of meaning itself. Such a view offers a remedy for the alienation and fragmentation of modernity. In a participatory universe, meaning is not imposed externally but arises organically through engaged awareness.

## Conclusion

The persistence of the Hard Problem of Consciousness is not due solely to the limits of neuroscience, but to the metaphysical assumptions that underlie dominant scientific paradigms. Transpersonal psychology, drawing from ancient contemplative wisdom, contemporary philosophy, and experiential exploration, offers a compelling alternative: consciousness is not a latecomer in the cosmic story but the very ground of being. By embracing this ontological reversal, we do not reject scientific inquiry but expand it. Rather than confining consciousness to neural correlates, we explore its role as a co-creative force in the unfolding of reality. This paradigm shift holds profound implications. It reorients psychology toward meaning, ethics, and the sacred. It offers a framework in which human suffering can be transmuted into growth and spiritual awakening. It invites us to recognize our embeddedness in a conscious, evolving universe.

In such a worldview, the study of consciousness is not merely an academic endeavor but a sacred vocation. It calls for a union of intellect and intuition, rigor and reverence, analysis and awe. As we move toward a science of consciousness that is inclusive of inner life, cultural wisdom, and spiritual insight, we come closer to a vision of reality that is not only more complete but more deeply humane.

## Data Availability

The original contributions presented in the study are included in the article/supplementary material, further inquiries can be directed to the corresponding author.
