# Correction: The spiritual core of the hard problem: consciousness as foundational, not emergent

**DOI:** 10.3389/fpsyg.2026.1804305

**Published:** 2026-03-25

**Authors:** Amira Arora

**Affiliations:** Independent Researcher, Trenton, NJ, United States

**Keywords:** consciousness is primary, transpersonal psychology, non-dual traditions, Advaita Vedanta, contemplative science, participatory knowing, spiritual phenomenology, cosmology and consciousness

The reference for “Kelly, 2016” was erroneously written as “Kelly, E. F. (2016). Consciousness and psi: implications for psychology. *J. Conscious. Stud*. 23, 9–23.” It should be “Kelly, E. F., Crabtree, A., and Marshall, P. (Eds.). (2015). *Beyond Physicalism: Toward Reconciliation of Science and Spirituality*. Lanham, MD: Rowman & Littlefield.”

The in-text citation was erroneously written as “(Ferrer, 2017; Kelly, 2016)” and should be “(Ferrer, 2017; Kelly et al., 2015)”. The correct citation has been inserted to section “Ontological reversals and participatory realism.”

The reference for “Vieten et al., 2020” was erroneously written as “Vieten, C., Langone, A., and Palmer, J. (2020). The neuroscience of spiritual practice: integrating ancient wisdom and modern science. *Front. Psychol*. 11:522. doi: 10.3389/fpsyg.2020.00522.” It should be “Vieten, C., Scammell, S., Pilato, R., Ammondson, I., Pargament, K. I., and Lukoff, D. (2013). Spiritual and religious competencies for psychologists. *Am. Psychol*. 68, 291–302. doi: 10.1037/a0032699.”

The in-text citation was erroneously written as “(Vieten et al., 2020)” and should be “(Vieten et al., 2013)”. The correct citation has been inserted to sections “**Introduction**” and “**The materialist paradigm and its limits**.”

The reference for “Kastrup, 2021” was erroneously written as “Kastrup, B. (2021). *The idea of the world: A multi-disciplinary argument for the mental nature of reality*. Amsterdam, Nertherlands: Springer.” It should be “Kastrup, B. (2019). *The Idea of the World: A Multi-Disciplinary Argument for the Mental Nature of Reality*. Winchester: Iff Books.”

The in-text citation for [Kastrup] was erroneously written as “(Goff, 2019; Kastrup, 2021)” and should be “(Goff, 2019; Kastrup, 2019)”. The correct citation has been inserted to sections “**The filter theory and transpersonal consciousness**” and “**The materialist paradigm and its limits**.”

The reference for “Vago & Zeidan (2016)” was erroneously linked to “Vago, D. R., and Zeidan, F. (2016). Mindfulness meditation and its impact on emotional well-being. *Psychol. Sci*. 27, 1334–1344. doi: 10.1177/0956797616632491” The correct reference is “Vago, D. R., and Zeidan, F. (2016). The brain on silent: Mind wandering, mindful awareness, and states of mental tranquility. *Ann. N. Y. Acad. Sci*. 1373, 96–113. doi: 10.1111/nyas.13171.”

The reference for “Greyson, B. (1983)” was erroneously linked to “Greyson, B. (2022). The near-death experience scale: construction, reliability, and validity. *J. Nerv. Ment. Dis*. 209, 539–197. doi: 10.1097/NMD.0000000000001397.” The correct link is “Greyson, B. (1983). The near-death experience scale: Construction, reliability, and validity. *J. Nerv. Ment. Dis*. 171, 369–375. doi: 10.1097/00005053-198306000-00007.”

The in-text citation for “Greyson, B. (1983)” was erroneously written as “(Greyson et al., 2022)”. A correction has been made to the sections “**Introduction**” and “**The materialist paradigm and its limits**.”

The reference for “Greyson, B., Bruce, R., and Miller, C. (2022). *The neurobiology of near-death experiences: an overview of recent research*. *Conscious. Cogn*. 99:103284.” does not exist; the article number/DOI point to a different paper. The citation has been removed from the section “References”.

The reference and respective in-text citation for “Koch, C., and Hepp, K. (2006). *The integrated brain: A theoretical model of consciousness*. Berlin: Springer.” does not exist; the article number/DOI point to a different paper. The citation has been removed from the sections “References” and “The materialist paradigm and its limits.”

The reference and respective in-text citation for “Marshall, P. (2021). *Psychedelic medicine: a re-emerging therapeutic paradigm*. *Br. J. Psychiatry, 219*, 383–391. doi: 10.1192/bjp.2021.18” does not exist; the article number/DOI point to a different paper. The citation has been removed from the section “References” and “Introduction, opening paragraph”.

The reference and respective in-text citation for “Nour, M. M., Carhart-Harris, R., and Ly, C. (2016). *The therapeutic potential of psychedelics in mental health treatment*. *Psychology of Consciousness: Theory, Research, and Practice*, 3, 207–223.” do not exist in the journal's archives. The citation has been removed from the sections “The materialist paradigm and its limits” and “References”.

The reference for “Yaden, D. B., Weng, H., and Harvey, B. (2017). *The study of consciousness in the context of spiritual experience*. *Frontiers in Psychology*, 8:1578.” was erroneous. The DOI provides a different article on EMDR therapy. The reference and the respective in-text citation have been removed from the manuscript sections “The materialist paradigm and its limits” and “References.”

The reference and respective in-text citation for “Castro (2014)” were removed from manuscript sections “Cultural implications and the need for a new metaphysical narrative” and “References” after verification revealed inconsistencies with the published record.

The reference “Metzinger, T., and Windt, J. M. (2016). *The philosophy of consciousness and its limits*. Cambridge, MA: MIT Press.” was erroneously listed in the bibliography. The reference has been removed from the manuscript sections “References” and “Introduction, opening paragraph.”

The reference and respective in-text citation for Charbonier, 2014 were removed from manuscript sections “The materialist paradigm and its limits” and “References” after verification revealed inconsistencies with the published record.

The reference and respective in-text citation for Hassel M8rch (2020) were removed from manuscript sections “A challenge to scientific orthodoxy” and “References” after verification revealed inconsistencies with the published record.

The reference and respective in-text citation for MacDonald, 2000 were removed from manuscript sections “Discussion” and “References” after verification revealed inconsistencies with the published record.

The reference and respective in-text citation for May et al., 2019 were removed from manuscript sections “The filter theory and transpersonal consciousness” and “References” after verification revealed inconsistencies with the published record.

The reference and respective in-text citation for Michel Bitbol (2011) were removed from manuscript sections “Discussion” and “References” after verification revealed inconsistencies with the published record.

The reference and respective in-text citation for Michel Bitbol (2014) were removed from manuscript sections “Ontological reversals and participatory realism” and “References” after verification revealed inconsistencies with the published record.

The reference and respective in-text citation for Roseman et al., 2018 were removed from manuscript sections “The filter theory and transpersonal consciousness” and “References” after verification revealed inconsistencies with the published record.

The reference and respective in-text citation for Shah-Kazemi, 2010 were removed from manuscript sections “Cross-cultural perspectives on primordial consciousness” and “References” after verification revealed inconsistencies with the published record.

The reference and respective in-text citation for Seth, 2021 were removed from manuscript sections “The materialist paradigm and its limits” and “References” after verification revealed inconsistencies with the published record.

The reference and respective in-text citation for Seth and Bayne, 2022 were removed from manuscript sections “Introduction” and “References” after verification revealed inconsistencies with the published record.

The reference and respective in-text citation for Tang et al., 2015 were removed from manuscript sections “Phenomenology and the intimacy of experience” and “References” after verification revealed inconsistencies with the published record.

The reference and respective in-text citation for Taylor, 2017 were removed from manuscript sections “Existential and ethical reflections” and “References” after verification revealed inconsistencies with the published record.

The reference and respective in-text citation for Walach et al., 2021 were removed from manuscript sections “Introduction” and “References” after verification revealed inconsistencies with the published record.

The reference and respective in-text citation for Walach, 2021 were removed from manuscript sections “Cross-cultural perspectives on primordial consciousness” and “References” after verification revealed inconsistencies with the published record.

The **Generative AI statement** was erroneously given as:

The author(s) declare that no Gen AI was used in the creation of this manuscript.

Any alternative text (alt text) provided alongside figures in this article has been generated by Frontiers with the support of artificial intelligence and reasonable efforts have been made to ensure accuracy, including review by the authors wherever possible. If you identify any issues, please contact us.

The correct **Generative AI statement** appears below:

The author(s) declared that generative AI was used in the creation of this manuscript. Artificial intelligence (AI) tools were used solely for basic grammar correction and language refinement in the preparation of this manuscript. Specifically, Grammarly was used to improve the readability and linguistic clarity of the English text. All scientific content, data interpretation, and conclusions were developed independently by the author.

Any alternative text (alt text) provided alongside figures in this article has been generated by Frontiers with the support of artificial intelligence and reasonable efforts have been made to ensure accuracy, including review by the authors wherever possible. If you identify any issues, please contact us.

The original version of this article has been updated.

